# Case Report: A renal wasting disease caused by a pure deletion of nephrocystin-1

**DOI:** 10.3389/fped.2025.1541411

**Published:** 2025-06-20

**Authors:** Ting Dong, Jiajia Luo, Tianhong Sun, Huimin Wu, Qing Zhao, Lina Ma, Jing Yang

**Affiliations:** ^1^ Department of Lanzhou University, Lanzhou, China; ^2^ Department of Pediatrics, Lanzhou University Second Hospital, Lanzhou, China

**Keywords:** renal wasting disease, *NPHP1* gene, pediatric, case report, sequencing of genome case report (presentation)

## Abstract

Nephronophthisis is an autosomal recessive disorder associated with the tubular interstitium of the kidney, and can lead to renal failure in children and adolescents. Mutations in the gene encoding nephrocystin-1, *NPHP1*, are frequently associated with the disease. Here, we describe the case of a child who presented to the clinic with febrile convulsions and who was ultimately diagnosed with nephronophthisis caused by a homozygous deletion of the *NPHP1* gene. Alerting pediatricians to the recognition of atypical renal wasting disease and reclarifying the diagnostic value of genetic diagnosis for this disease.

## 1 Introduction

Nephronophthisis (NPHP) is recognized as the most common inherited cause of renal failure in children (1). According to the age of onset of end-stage renal disease (ESRD), NPHP can be classified as infantile, juvenile and adolescent forms. Among them, juvenile NPHP is the most common. Typical clinical features include polyuria, polydipsia and isosthenuria due to renal concentration function impairment, no hematuria, proteinuria, hypertension and other manifestations. If the disease progresses to a higher stage of chronic kidney disease (stage III and above), fatigue may occur. Fever, fatigue, skin itching, nausea, vomiting, uremic gastritis, anemia, and growth retardation may occur (2). Approximately 10%–20% of cases involve NPHP patients also display additional extrarenal abnormalities, such as Senior-Loken syndrome (SLS), Joubert syndrome, Mainzer-Saldino's syndrome, and liver fibrosis, situs inversus, etc. Among these, the eye is the most commonly affected extrarenal organ in NPHP, with retinitis pigmentosa being the most common (3).

Genes that have been found to be associated with the disease encode proteins involved in the functioning of primary cilia, basal bodies, and centrosomes, and mutations can lead to renal damage, as well as extrarenal manifestations associated with NPHP. Mutations in the nephrocystin-1 gene (*NPHP1*) on chromosome 2q13, the most common cause of the disease, are either pure or compound heterozygous mutations (4). In this report, we describe the case of a child with renal wasting disease caused by a pure deletion of *NPHP1* and analyze and summarize the manifestations of NPHP in this child, to provide a reference for the diagnosis and treatment of similar children in the future and to enhance the understanding and identification of the disease in clinical practice, thus providing timely diagnosis and treatment.

## 2 Case report (presentation)^1^

### 2.1 Medical history

The child was 6 years and 9 months old and presented to the clinic with “one day of fever with two convulsions”. The convulsions were characterized by loss of consciousness, bluish lips, generalized twitching, rigidity of the limbs, no incontinence, and foaming at the mouth; these lasted for two minutes and then subsided before starting again with the same manifestations and lasting between three and four minutes. After an interval of approximately 30 min, the convulsions began again. Cranial nuclear magnetic resonance imaging (MRI), electroencephalography (EEG), laboratory tests (see Table 1), and genetic tests were performed. The diagnosis was renal wasting disease, stage 4 renal impairment, secondary hyperparathyroidism, renal anemia, and renal tubular damage. The child was given human erythropoietin 2,000 IU/session once a week, as well as levetiracetam, uremic granules, capsules of aldoxyl starch, compound a-ketoacid tablets, vitamin D drops, and other medications, and was followed up regularly for three months, with advice to undergo kidney transplantation in case of deterioration of renal function.

**Table 1:** Pertinent laboratory results.

Parameter (unit)	May 29th	June 17th	July 06th	July 28th	August 19th	Reference range
Blood–RT						
RBC (10^12^/L)	2.63↓	3.14↓	3.15↓	3.22↓	3.2↓	4.2–5.7
HGB (g/L)	73↓	89↓	88↓	95↓	87↓	118–156
HCT (L/L)	0.226↓	0.276	0.279↓	0.285↓	0.285↓	0.36–0.46
Urine–RT						
Uβ2MG (μg/L)	9,220↑	9,342↑	10,523↑	9,742↑	9,393↑	<200
URBP (mg/L)	18.06↑	19.83↑	27.48↑	25.07↑	26.03↑	<0.7
UM/UC (mg/mmol)	10.0↑	10.7↑	9.7↑	11.8↑	12.3↑	≦3
Serum chemistry						
ALT (U/L)	5↓	6	4↓	7	7.2	7–30
ALP (U/L)	150	158	146	117↓	109↓	143–406
UREA (mmol/L)	20.52↑	24.10↑	24.50↑	26.8↑	27.71↑	2.7–7.0
CREA (μmol/L)	263.4↑	290.4↑	294.6↑	305.4↑	307.7↑	22–66
CO_2_ (mmol/L)	13.9↓	16.8↓	21.5↓	15.1↓	20.8↓	22–29
PTH (μg/ml)	743↑	534↑	254↑	—	—	11.00–67.00

### 2.2 Antecedent history

The patient was in good health, with no symptoms of polyuria, thirst or renal dysfunction. The patient denied history of surgery, trauma or blood transfusion. The patient denied close contact with infectious diseases such as hepatitis and tuberculosis, and denied food and drug allergies.

### 2.3 Family history

**Figure 1:**
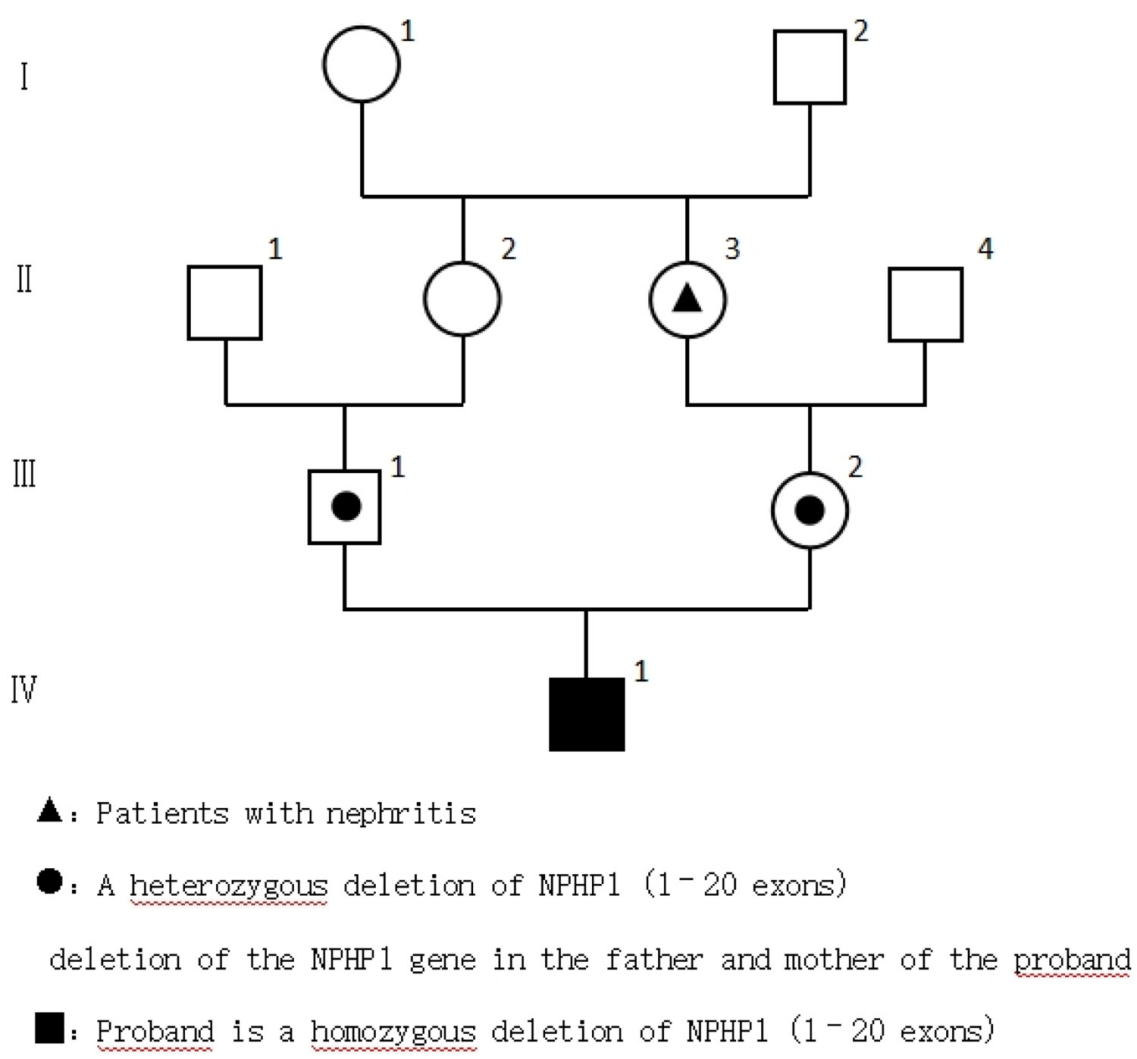
Genealogical chart of the child.

The parents were healthy, the mother had one pregnancy and no abortion, and the marriage was close relatives. The family history of hereditary diseases was denied. The maternal grandmother had a history of nephritis (Figure 1).

### 2.4 Personal history

First pregnancy, first delivery, with full-term normal delivery. The birth weight was 3.3 kg and the mother is health during pregnancy.

### 2.5 Laboratory results

**Figure 2:**
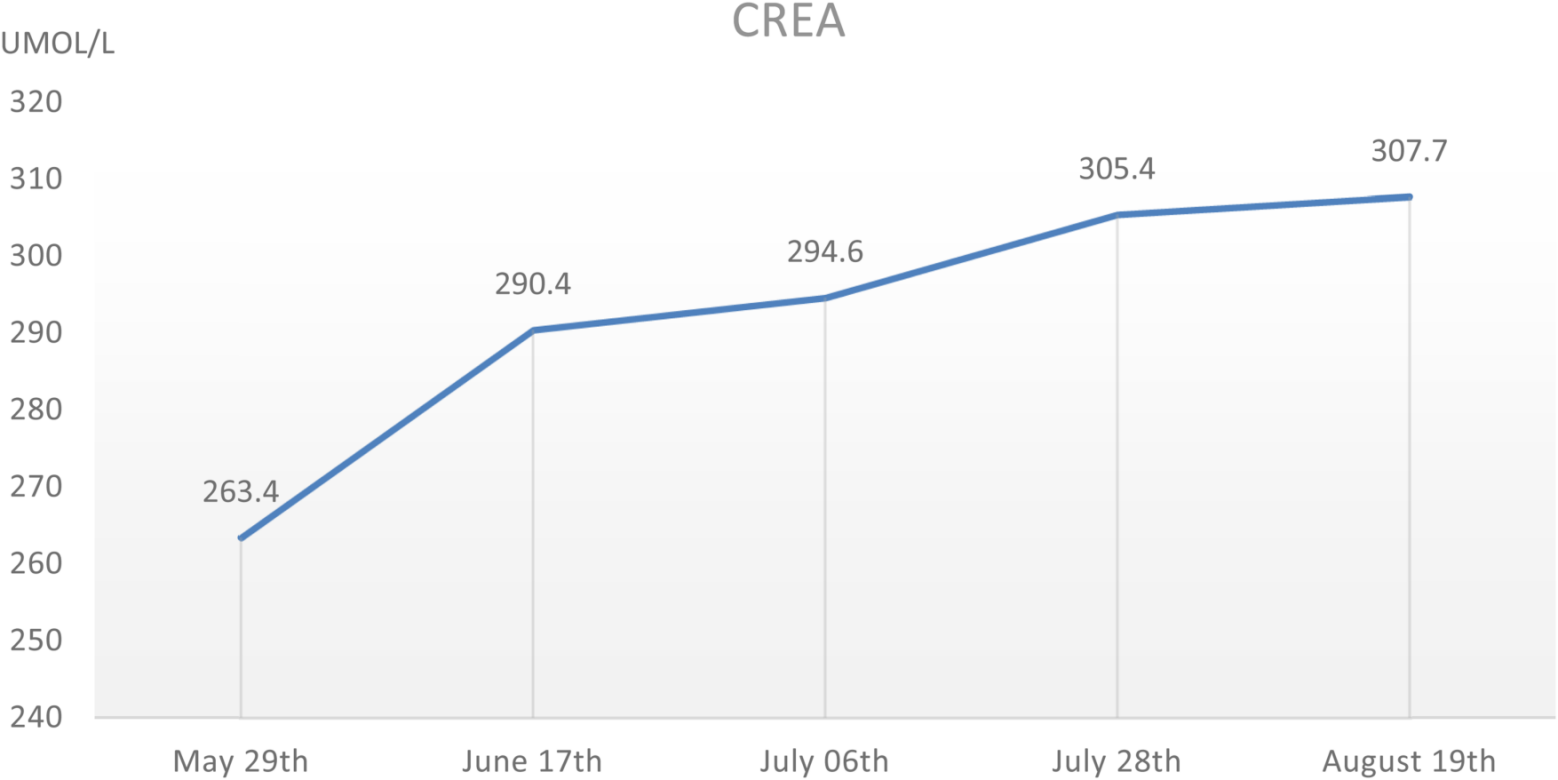
The CREA level of this child during hospitalization, which showed a gradual increase.

**Figure 3:**
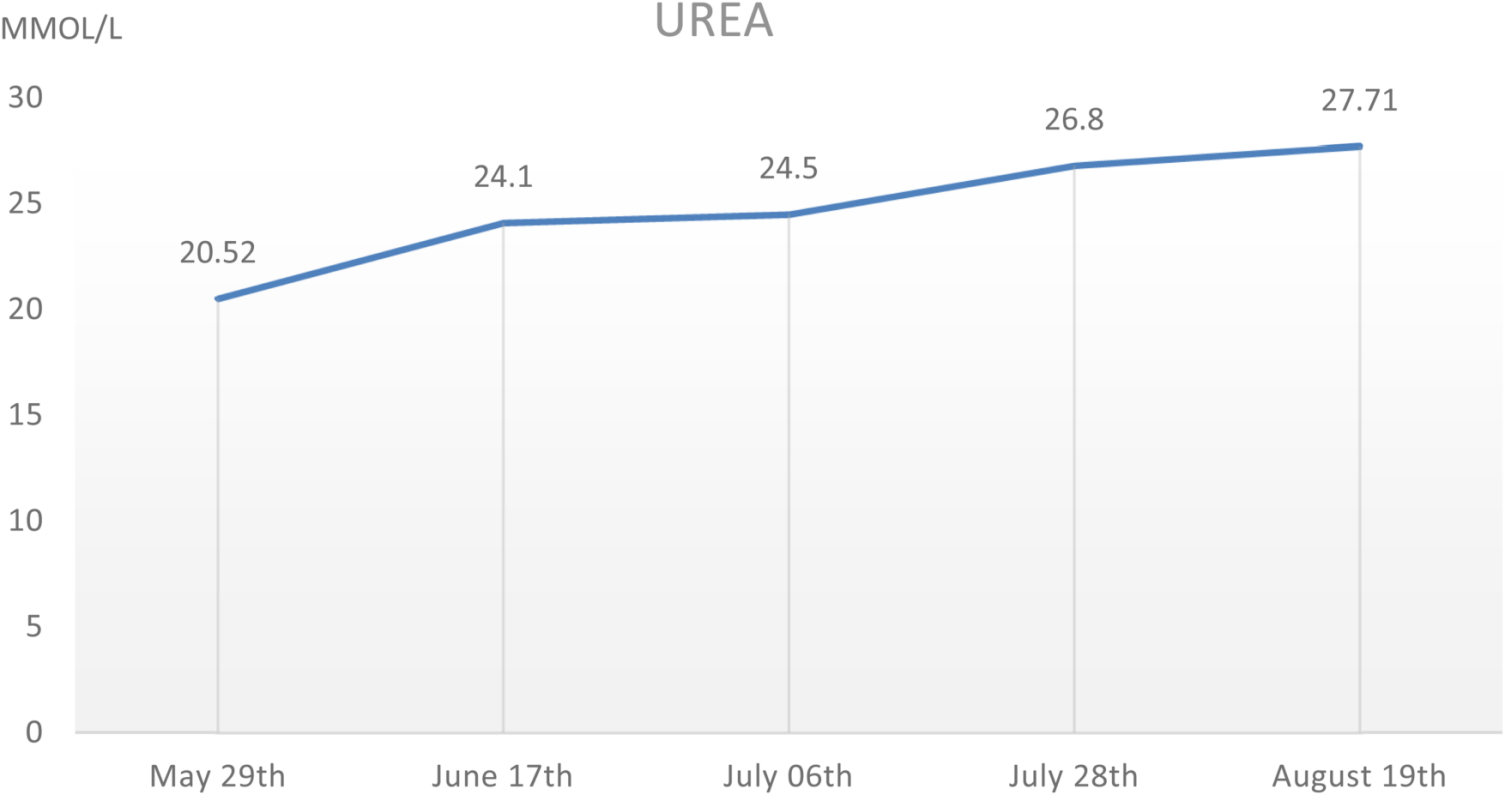
The UREA level of this child during hospitalization, which showed a gradual increase.

During the hospitalization period, the level of relevant renal function indexes (Urine-RT, UREA, CREA, etc.) was significantly higher than the normal level (Figures 2,3), showing an upward trend, which made it clear that the child's renal function was impaired, and the blood routine was improved, suggesting that: the red blood cell count and hemoglobin were lower than the normal level, which was secondary renal anemia, and the child was given active treatment with human erythropoietin, and the level of the child's parathyroid hormone was significantly elevated. At the same time, the parathyroid hormone level of the child was found to be significantly elevated, which was considered to be secondary hyperparathyroidism, in line with the secondary manifestation of NPHP.

### 2.6 Imaging results

#### 2.61 MR urinary tract plain scan + MRU (including kidney, ureter, and bladder)

**Figure 4:**
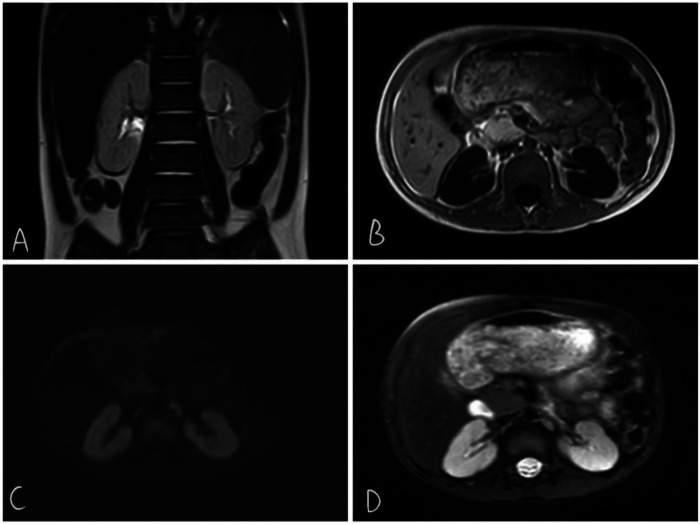
T2wi coronal view shows somewhat poorly defined corticomedullary boundaries in both kidneys **(A)** T1WI shows long TIWI signals in both kidneys **(B)** DWI shows no restriction of diffusion **(C)** T2WI signal shows slightly increased cortical signal in T2WI **(D)**

Diagnostic opinions: (1) T1WI showed slightly reduced and diffuse bilateral renal signals, with an unclear boundary between the cortex and medulla. (2)Mild expansion of the middle ureter on both sides (Figure 4).

#### 2.62 Other examinations

(1) Abdominal color Doppler ultrasound: Diffuse lesions in both kidneys, no abnormality was detected in the liver, gallbladder, pancreas and spleen.
(2) Echocardiography: Patent foramen ovale (occasionally visible).
(3) Fundus color Doppler ultrasound: binocular ametropia, binocular amblyopia.
(4) No abnormality was found on EEG.
(5) MR brain stereotactic + intraoperative navigation + epileptic sequence scan (large-scale fine scan): No obvious abnormality was found in the brain stereotactic scan.

#### 2.67 Genetic testing

**Figure 5:**
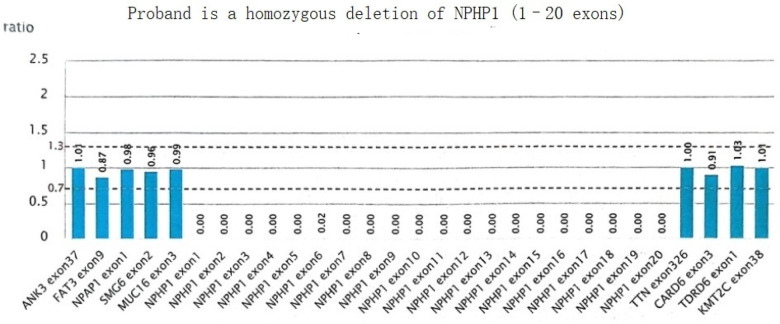
NPHP1 gene sequencing results in subjects.

A homozygous deletion variant in the *NPHP1* gene was detected: [NM_001128178.3(NPHP1):loss2(EXON:1-20)(all)], resulting in homozygous deletion of exon 1 to exon 20 of the *NPHP1* gene, and which was suspected to involve the homozygous deletion of the entire gene (Figure 5).

**Figure 6:**
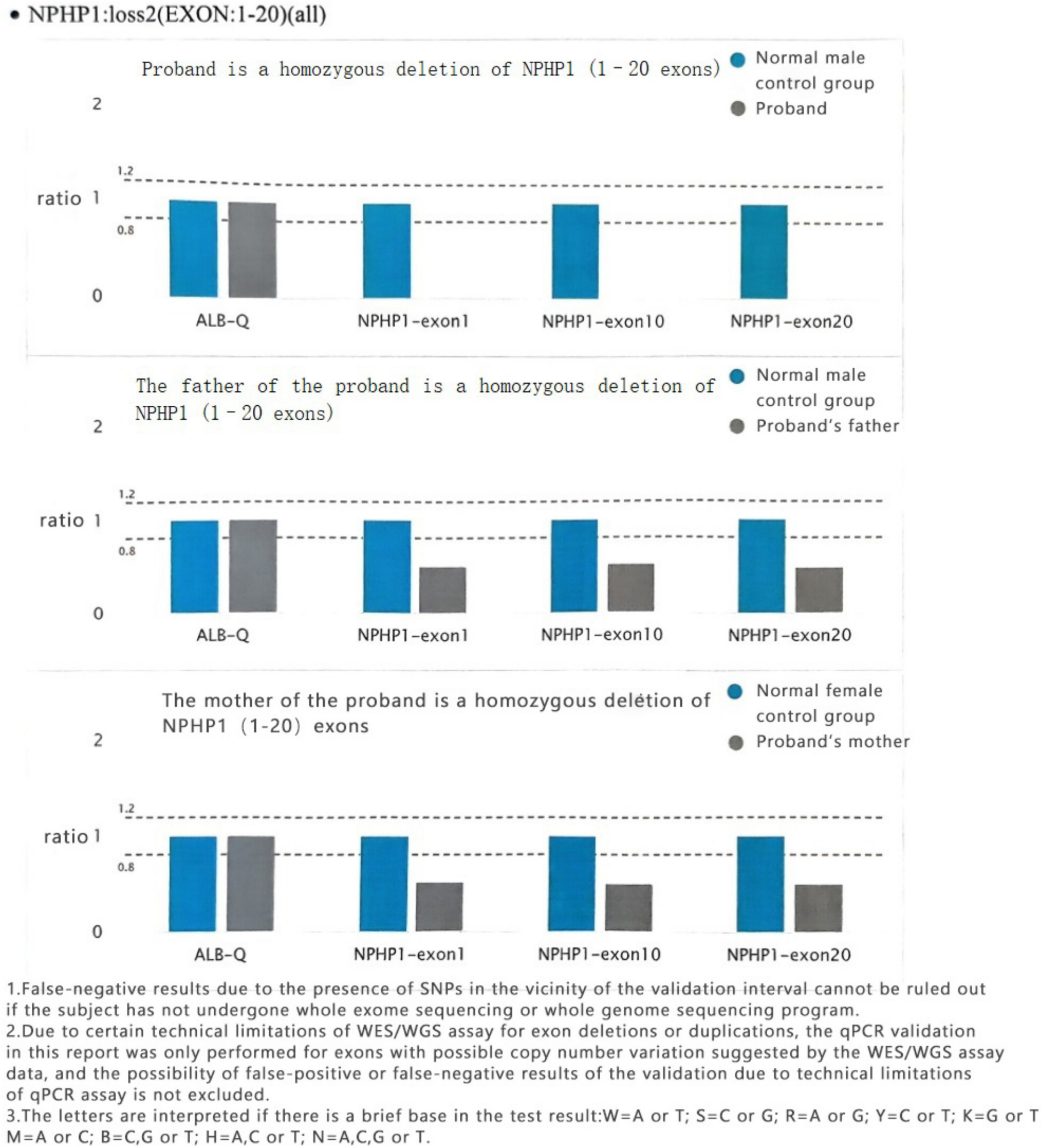
Sequencing results of the NPHP1 gene in the subject's parents.

Both parents of the subject had loss of heterozygosity in *NPHP1* (EXON:1–20) (Figure 6).

#### 2.68 Diagnostic

In this case, the child was admitted to hospital due to sudden convulsions. However, no obvious abnormalities were found on neurological examinations. During hospitalization, high levels of serum creatinine and urea nitrogen were found, although the cause of the renal dysfunction was unknown. After evaluation, the child was diagnosed with kidney impairment stage 4. Typical symptoms of renal wasting disease had not yet appeared. And ultrasound examination only indicated diffuse lesions in both kidneys. The parents of the child were consanguineous, and genetic sequencing was performed to identify the cause of the renal impairment. The sequencing results identified a homozygous deletion of the *NPHP1* gene. And the diagnosis thus requires differentiation from medullary cystic nephropathy, early autosomal dominant polycystic kidney disease (PKD), autosomal recessive PKD, and acquired tubulointerstitial injury. Although these diseases overlap in some features, they can be distinguished by genetic testing and renal biopsy (5). Infantile NPHP typically presents with ESRD before the age of 4, characterized by NPHP interstitial fibrosis, tubular atrophy, and extensive cyst formation similar to polycystic kidney disease. Due to the widespread formation of renal cysts, the kidneys appear not significantly reduced in size or even enlarged on ultrasound. In this case, the patient developed ESRD at age 6 and is not considered to have infantile NPHP (6).

This, together with the extrarenal manifestations of renal depletion disease (convulsions, decreased visual acuity in both eyes, secondary hyperparathyroidism), results of auxiliary examinations, genetic testing and differential diagnosis, led to the diagnosis of juvenile NPHP.

## 3 Discussion

Nephronophthisis (NPHP) is one of the most common causes of monogenic single-gene renal failure (CKF), with over 25 genes have been associated with NPHP. These genes encode proteins involved in the functioning of primary cilia, the matrix, and the centrosome, leading to kidney diseases and extrarenal manifestations (7, 8). Among them, the most common cause of juvenile NPHP is a mutation in the *NPHP1* gene, which accounts for 20%–25% of reported NPHP cases; 10 cases of renal wasting disease have been found to be caused by homozygous deletion of a large fragment of *NPHP1*, with the onset of the disease occurring at around 9–13 years old (9). Due to renal concentrating function, most patients exhibit typical symptoms of nephronophthisis, such as polydipsia, polyuria, enuresis, and significantly decreased urine osmolality. Moreover, ultrasound examinations of patients with nephronophthisis caused by NPHP1 gene deletion have consistently shown renal cysts, but the child in this case only suggests that the echo of the parenchyma of both kidneys is enhanced, the boundary between cortex and medulla is blurred, and the blood flow of both kidneys is less.

Renal cortical cysts may appear in the late stage of the disease. These are primarily caused by the NPHP1 protein (also known as renal capsule protein) which is expressed on the surfaces of primary cilia and renal tubular epithelial cells, and can interact with molecules involved in cell adhesion and signal transduction, as well as other capsule proteins. Fibronectin is an important component of the extracellular matrix and is involved in cell proliferation, apoptosis, and polarization (10). Mutations in *NPHP1* can prevent its binding to fibronectin, thus restricting its function. Apart from the appearance of a series of extrarenal manifestations, renal cysts develop as the disease progresses. It has been reported that the initial imaging results of patients with NPHP may be non-specific, while the normal shape of the kidney can be assessed by ultrasound, indicating a loss of corticomedullary differentiation. However, with disease progression, ultrasound imaging conducted at later stages may show smaller atrophic kidneys with obvious corticomedullary cysts (11). Therefore, it is thus important to pay active attention to the manifestations associated with renal cysts in such children.

Renal tubulointerstitial damage in renal wasting disease is often characterized by medullary cystic changes. Myelocystic nephrotic include juvenile renal wasting disease (JN), medullary cystic kidney disease (MCKD), and medullary sponge kidney (MSK) (12). MSK is a kind of congenital, benign renal developmental abnormality, primarily characterized by spindle-shaped or small cystic dilation of the collecting ducts within one or more renal pyramids in both kidneys. Clinical manifestations often include kidney stones, renal calcification, tubular concentrating dysfunction, and recurrent urinary tract infections. The prognosis is relatively good, with rare cases causing renal function abnormalities during adolescence. Ultrasound (ultrasound examination) may show small anechoic areas and echogenic foci radiating around the renal pelvis. In this case, no relevant ultrasound findings were observed in the patient's kidneys, so this diagnosis is not considered for now. JN and MCKD share similarities in clinical and pathological manifestations, collectively referred to as the JN-MCKD syndrome. JN-MCKD is a group of cystic kidney diseases characterized by the formation of medullary cysts and occult chronic renal insufficiency, with pathological features consistent with chronic tubulointerstitial nephritis. The two can be distinguished based on inheritance pattern, age of onset, and clinical presentation: renal wasting disease is an autosomal recessive disorder, commonly seen in children and adolescents, and may be accompanied by other extrarenal manifestations. Medullary cystic disease, which is more common in adults, is an autosomal dominant inheritance, mainly with renal lesions and less extrarenal manifestations. Both are genetic diseases, which are often diagnosed by genetic testing.

Feng Chen mentioned that the clinical diagnosis of atypical NPHP is challenging, proposed NPHP1 deletion in Chinese twins with nephronophthisis, and demonstrated in the text the superiority of whole exome sequencing (WES) for diagnosing this type of disease (13). This case patient is also an atypical NPHP patient. Given that the average age of onset of ESRD in juvenile NPHP is 13 years, with only a few cases entering ESRD around age 6, the diagnosis of this patient is even more difficult. This reaffirms the importance of genetic testing for diagnosing atypical NPHP patients.

## Data Availability

The original contributions presented in the study are included in the article/Supplementary Material, further inquiries can be directed to the corresponding author.

## Footnotes

^1^Project supported by the Nature Science Foundation of Gansu Province, China (No. 22JR11RA052)
